# Supplementary material for: Splice-Junction-Based Mapping of Alternative Isoforms in the Human Proteome
Source: Cell Rep. Author manuscript; Available in PMC 2020 Jan 15. (PMC6961840; doi:10.1016/j.celrep.2019.11.026)
Supplement: 2 [file NIHMS1546469-supplement-2.zip › DF1/PXD006675/CardiacFibroblast/CardiacFibroblast_5_PALLD_EDLLNNGQPIYWFK.pdf]

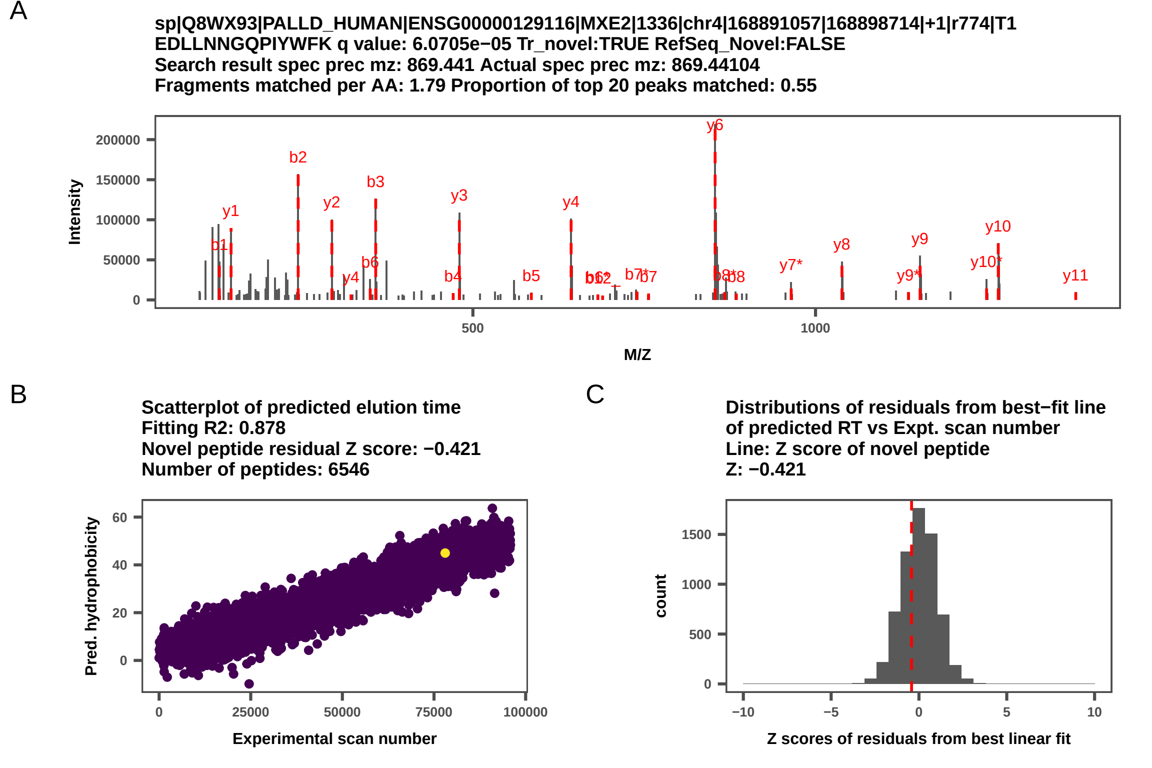
A
sp|Q8WX93|PALLD_HUMAN|ENSG00000129116|MXE2|1336|chr4|168891057|168898714|+1|r774|T1
EDLLNNGQPIYWFK q value: 6.0705e−05 Tr_novel:TRUE RefSeq_Novel:FALSE
Search result spec prec mz: 869.441 Actual spec prec mz: 869.44104
Fragments matched per AA: 1.79 Proportion of top 20 peaks matched: 0.55
Intensity
200000
150000
100000
50000
0
500
1000
M/Z
y1
b1
b2
y2
y4
b6
b3
y3
b4
b5
y4
y6
y7*
y8
y9
y9*
y10
y10*
y11
B
Scatterplot of predicted elution time
Fitting R2: 0.878
Novel peptide residual Z score: −0.421
Number of peptides: 6546
Pred. hydrophobicity
60
40
20
0
0
25000
50000
75000
100000
Experimental scan number
C
Distributions of residuals from best−fit line
of predicted RT vs Expt. scan number
Line: Z score of novel peptide
Z: −0.421
count
1500
1000
500
0
−10
−5
0
5
10
Z scores of residuals from best linear fit
